# Confrontation of AlphaFold models with experimental structures enlightens conformational dynamics supporting CYP102A1 functions

**DOI:** 10.1038/s41598-022-20390-6

**Published:** 2022-09-25

**Authors:** Philippe Urban, Denis Pompon

**Affiliations:** Toulouse Biotechnology Institute, CNRS, INRAE, INSA, Université de Toulouse, 135 Avenue de Rangueil, Toulouse, France

**Keywords:** Biochemistry, Chemical biology, Computational biology and bioinformatics, Structural biology

## Abstract

Conformational dynamics plays a critical role for the function of multidomain electron transfer complexes. While crystallographic or NMR approaches allow detailed insight into structures, lower resolution methods like cryo-electron microscopy can provide more information on dynamics. In silico structure modelling using AlphaFold was recently successfully extended to the prediction of protein complexes but its capability to address large conformational changes involved in catalysis remained obscure. We used bacterial CYP102A1 monooxygenase homodimer as a test case to design a competitive modelling approach (CMA) for assessing alternate conformations of multi-domain complexes. Predictions were confronted with published crystallographic and cryo-EM data, evidencing consistencies but also permitting some reinterpretation of experimental data. Structural determinants stabilising the new type of domain connectivity evidenced in this bacterial self-sufficient monooxygenase were analysed by CMA and used for in silico retro-engineering applied to its eukaryotic bi-component counterparts.

AlphaFold modelling of protein structures significantly surpasses the performances of previously described computational methods^1–3^. The approach was recently extended to protein complex with an efficiency as high as that of classical docking approaches^4^, including for high throughput screening of protein interactomes^5^. A development version of the algorithm named AlphaFold2_advanced (AF2A) constitutes an interesting alternative to the more finalized “multimer” version dedicated to complexes^6^, being faster and facilitating access to a range of possible alternate structures. Such models generally represent close conformers that could be averaged or sorted using AF2A-embedded or other tools exploiting structural conformity checking or energetic criteria^7^. Algorithm predictions can in some instances be influenced by biasing the AF2A-embedded multiple sequence alignment step, which can play a determining role in the selection among possible alternate structures^8^. However, prediction of the alternate folds experimentally characterized for fold-switching proteins was to date unsuccessful^9^, bringing doubt about AlphaFold capability to address markedly different conformations.

A situation of particular interest is that of modular protein complexes in which modules (i.e. domains) can compete together to associate into alternate geometries. The bacterial CYP102A1 from *Priestia megaterium*, a multi-modular cytochrome P450 (P450) that catalyses NADPH-dependent fatty acid hydroxylation^10^ was considered as a test case. This enzyme constitutes a dimer, in which each monomer folds into three independent modules: a C-terminal FAD containing domain binding NADPH (FADd), a FMN binding domain acting as electron carrier (FMNd) and an N-terminal heme-containing catalytic domain (P450d). The structural organization of the reductase part (consisting of both FADd and FMNd) is similar to that of eukaryotic NADPH-cytochrome P450 oxidoreductases (CPRs)^11,12^, assimilatory sulfite reductase^13^, methionine synthase reductase^14^, and nitric oxide synthases^15^. In bacterial CYP102A1 the reductase domain is fused at its N-terminus with a P450 domain^16^, when these two parts are separated and constitute a bi-component system in eukaryotes^17^. In the CYP102A1 dimer, each FMN domain must alternatively form an electron transfer competent complexes with one or the other of the two FAD and of the two P450 domains, leading to four possible and mutually exclusive geometries. Several reports based on CYP102A1 site-directed mutants and their biochemical analysis led to the dominant opinion that the electron transfers involved exclusively interchain reactions facilitated by the dimeric structure^18,19^. In addition, extensive analysis of chemical reticulation patterns by mass spectrometry brought some light on the intermonomer organization by identifying putative domain contacts^20^. Partial CYP102A1 crystal structures have been reported, including that of the isolated P450d^21^ and of the isolated FADd^22^. A fragment of CYP102A1 encompassing both P450d and FMNd was also crystallized and the structure of the resulting intrachain complex resolved^23^. In contrast, no crystal structures for the full-length protein, for the isolated reductase domain, or for any domain association in the dimer is available. However, low resolution imaging of a point mutant and higher resolution imaging of a synthetic variant featuring a shortened P450d-FMNd linker allowed acquisition of cryo-EM data at a sufficient resolution to embed previously available crystallographic structures into EM density maps leading to a global model for the dimer^24^.

Here, the CYP102A1 test case offered us the opportunity to evaluate conditions in which AF2A can predict ab initio for the same protein complex (i.e. CYP102A1 dimer) the alternate domain configurations required for catalysis. In this case and when confronted to conflictual modelling, alternate instantiations or differences in arbitrations by the AF2A self-attention algorithms resulted in prediction of markedly different conformations from run to run. Considering that AF2A does not explicitly involve energetic calculation but is mostly based on geometric pattern recognition, we decomposed the whole problem into a set of competitions between alternate modelling of ternary systems in which one partner can form exclusive complexes between two alternate other partners. Algorithm arbitrations based on geometric criterion are not expected to directly mirror relative thermodynamic stability of alternatively predicted complexes. However, geometric complementarities and resulting interaction energies at structural domain interfaces are somehow linked parameters^25,26^. In a similar approach, Saldano et al. have recently published a study on the accuracy with which AF2A in conjunction with ColabFold predicts the holo form of the protein bound to a biologically relevant ligand in a hand-curated collection of apo-holo pairs of conformers^27^. The results presented in this work illustrated that frequency of alternate arbitrations surprisingly correlates with expected changes in relative thermodynamic stabilities of alternate predicted structures and can also be used to direct rational engineering.

## Methods

### Softwares

AF2A modelling was performed using the Aphafold2_advanced^28^ Python notebook that was run on Google Collaboratory cloud computing facilities using GPU and its large (48 Go) dedicated memory. Google Colab notebooks^29^ were run using default parameters, except for the selection of the pTMscore ranking procedure, and the num_models parameter that was adjusted between 5 and 20 depending on cases. Resulting models were visualized and analysed using UCSF ChimeraX^30^, developed by the Resource for Biocomputing, Visualization, and Informatics at the University of California, San Francisco, and PyMOL molecular graphics system (Schrödinger). Matchmaker and ISOLDE plugins in ChimeraX were used for RMSD minimization and for model docking into cryo-EM density maps, respectively. AliView algorithm was used for sequence manipulations and visualization^31^ and the MIA-Muscle plugin for sequence alignments. TCoffee^32^ from Expresso web server was used for structure-based sequence alignments and the PRODIGY^33^ web server was used to calculate the binding free energies (ΔG) of interacting domains. ConsRank and CoCoMaps web applications were used respectively to rank models of complexes and to generate interaction maps of contacting residues^34^.

### Selection of models among alternate AF2A predictions

Each predicted structure (from 5 to 20) was examined. Structures in which the proteins were non-interacting (predicted binding energy lower than 3 kcal/mol) or featuring relative geometries inconsistent between repeated AF2A runs were considered to sign the absence of any predicted complex. Modeling of randomly overlapping structures between partners was also a frequent AF2A artifact signing the absence of defined complex. When formation of a defined complex was predicted, the AF2A recommended pTM score (in fact not the values but their ranks among models as illustrated on Supplementary Table 1) was not systematically consistent with calculated binding affinities using PRODIGY. In that case, the retained structure was based only on this last energetic criterion (i.e. the best affinity). When alternate geometries were predicted, the optimal fold for each was selected as previously. Formation of a dimeric structure was considered significant when predicted in at least 20% of the AF2A runs.

### Model refinements

AlphaFold models are by default unrelaxed and not refined to minimise the free energy of side chain conformation. Such refinements were found to marginally impact predicted main chain conformation and geometry of the protein–protein complex but could significantly alter calculation of presented interdomain binding free energies performed with PRODIGY software. Supplementary Table 1 compares the PRODIGY calculated interface binding free energies using best AF2A models both unrelaxed and following side chain conformation refinement using the Rosie software suite of Rosetta.

### Whole model reconstruction

Dimers of P450d, P450d-FMNd, and FADd-FMNd were modelled independently by using 5 to 10 AF2A modelling assays in each case. The binding free energies between polypeptide chains in each generated model were calculated using the PRODIGY software and the model featuring the higher binding free energy was conserved. Attempts to directly model the isolated FADd dimer were systematically unsuccessful, generating folded monomers or dimers with highly variable interfaces and low affinity. To bypass this, we modelled the FADd-FMNd dimer then we erased atom coordinates of the FMNd domain keeping coordinates for the FADd and the FADd-FMNd linker region unchanged.

Once all CYP102A1 parts modelled, these parts were manipulated as rigid bodies and not subjected to further adjustment during subsequent steps. For reconstruction of the full dimer structures, the two considered partial structures (either P450d-FMNd and FMNd(erased)-FADd dimers or P450d and FMNd-FADd dimers) were first moved in a way that the symmetry axis of the P450d (or the P450d-FMNd) dimer became aligned with the symmetry axis of the FMNd-FADd (or FADd) dimer. Once axes were aligned and merged, the distance between barycenter of the two sub-models along this common symmetry axis was adjusted to the shorter value not creating structural clashes. Then, the optimal rotation angle along this axis for the two sub-models cannot be determined directly by ab initio modelling due to the absence of direct contact between the FADd and P450d. Considering that the FMNd must alternatively form complexes with the P450d and FADd, it was hypothesized that the transition between the two resulting alternate structures must involve minimal conformation changes to optimize catalytic efficiency. Consequently, the dihedral angle between the FADd dimer and the P450d dimer models was chosen to a common value compatible with both the open and closed configurations without creating structural clashes. Alternatively embedding of modelled electronic density into EM density map was used to set this angle. However, both approaches gave rise to similar solutions within the error range. The structure intermediates required for the transition between the closed and the open conformations were not considered. Minor structural clashes that could be solved by side chain rotations at interfaces were optionally solved by applying molecular dynamics approach. The hybrid CYP102A1 model (one monomer in closed conformation, the other monomer in open conformation) was generated by partial substitution between the closed model and the open model following RMSD fit of the two models restricted to P450d and FADd matches. Models in the open conformation involving crossed (*trans*) and non-crossed (*cis*) geometries of FMNd-P450d dimers were generated in the same way. Chain continuity between partial models could be carried out by reestablishing broken peptide links followed by local chain conformation remodelling by energy minimisation. However, this step was not included in the presented models in which chain continuity was always checked to be feasible.

### Docking of AF2A models in the cryo-EM density maps

Cryo-EM density maps were filtered to a density level mostly eliminating the solvent noise when fitting of the global shape of models was attempted. However, when more precise structural details were targeted, filtering was increased to the higher level improving the resolution without visually impacting polypeptide chain continuity. These filtering levels can be variable, depending on the map considered and its resolution, and on the region targeted due to inhomogeneous levels of experimental map resolutions. ChimeraX tools and its ISOLDE plugin were then used to optimally define the dihedral angle along the common symmetry axis between the FADd and the P450d dimers using the experimental EM density map as a reference. During this process, structure fitting of the FMN domain into the EM density map was not considered to be a relevant criterion due to the probable contribution of experimental densities resulting simultaneously both from the open and closed conformations in the experimental EM map. Consequently, positioning of FMNd was determined based on the P450d density fitting for the conformation involving the P450d-FMNd complex and based on FADd density fitting for the conformation involving the FADd-FMNd complex.

### Complementary information

Cofactors are not currently modelled by AlphaFold. In all presented cases, FAD, FMN or heme cofactors were placed into modelled structures using the corresponding available crystal structures as references (PDB-4kew for heme, PDB-1bvy for FMN, and PDB-4dqk for FAD). For that, corresponding parts of modeled structures were structurally matched by RMSD minimisation with crystallographic structures and the cofactor atom coordinates transferred.

The method of structure reconstruction used to build the open-*cis* and the open-*trans* models given in Fig. 4 does not permit to model part of the linker connecting FADd to FMNd. Geometry of this part is expected to differ from the one in the closed model due to the need of rotation of the FMN domain to form the complex with the P450 domain. We checked that the loop length will be sufficient for connection but were unable to predict its exact conformation. Consequently, the corresponding 20-residue sequence segment (spanning from SDVA to NKST) was erased from models in Fig. 4c–d. Additionally, the presented open-*trans* model obtained using reconstruction hypothesis is not completely satisfactory at the level of the FMNd-FADd contact due to some residual side chain clashes that cannot be resolved while maintaining a good docking into the cryo-EM maps. This model must thus be considered as only indicative in contrast to the better open-*cis* model. Figure 5d and Supplementary Figure S3a (right part) were, for comparison purpose, copied with some reformatting from the discussed cryo-EM publication due to the non-availability of corresponding coordinates to rebuild figures. Due to limited sequence similarities the sequence alignment presented in Fig. 6 were based on common structural motifs. Relative alignment of the groups of bacterial and eukaryotic sequences is thus approximate due to the lack of sufficient similarity. The structural overlay of Fig. 6a (right panel) was obtained by RMSD minimisation restricted to FADd. Common β-sheet motifs used for alignments are visible on the right and left part of the engineered sequences described in the remaining of the figure.

## Results and discussion

### Prediction of alternate structures of CYP102A1

**Figure 1:**
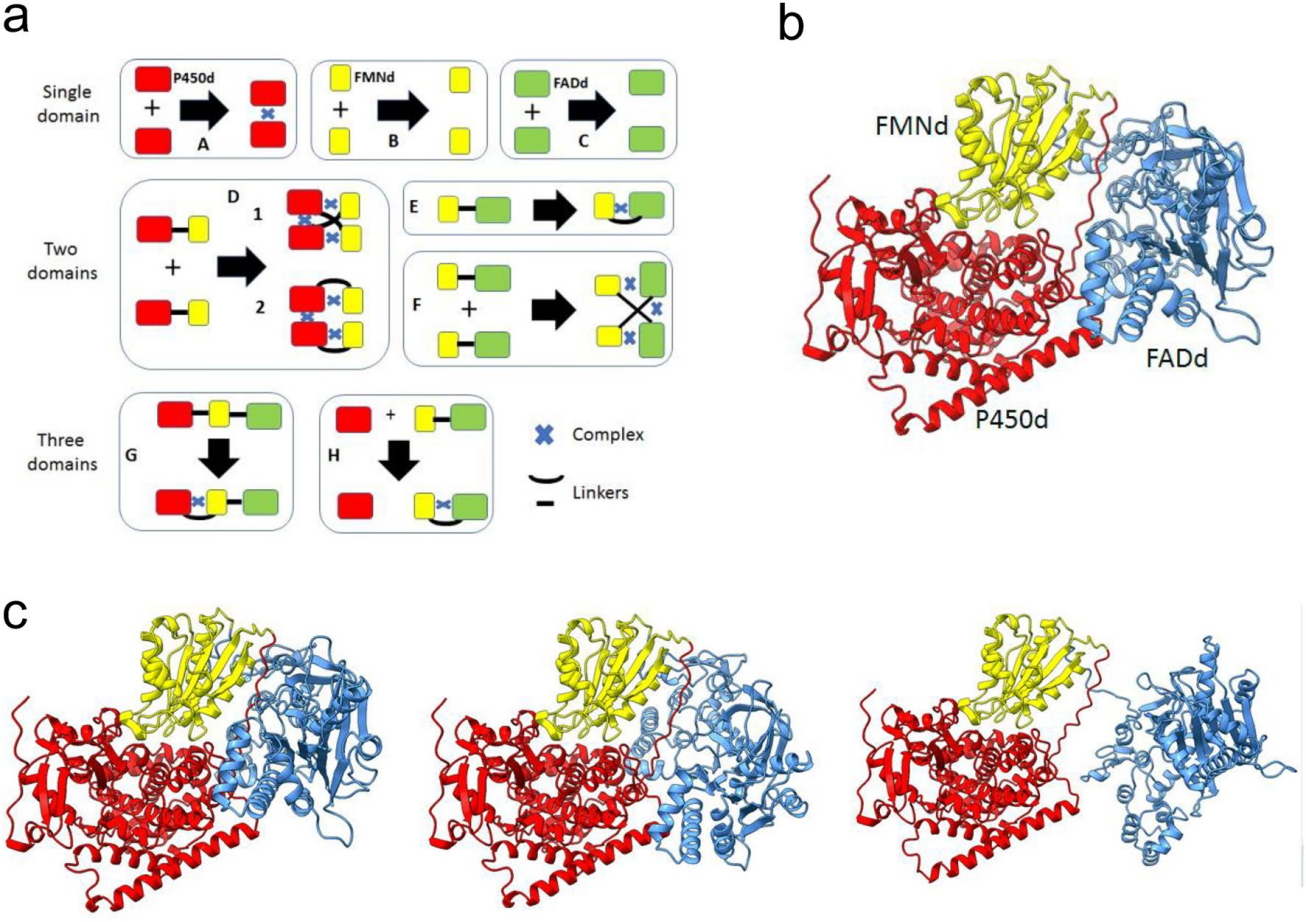
Modeling of CYP102A1 monomer and of partial structures. (**a**), Partial models considered for the assembly of the full-length CYP102A1 dimer structure. P450d, FMNd and FADd are represented as red, yellow and green boxes, respectively. Polypeptide chain linkers are indicated by solid lines and the AF2A-predicted complex formations with a blue cross. (**b**), Model for CYP102A1 monomer released in AlphaFold Protein Structure Database (entry P14779). P450d, FMND, and FADd are colored red, yellow, and blue, respectively. (**c**), Alternate AF2A predictions obtained upon repeated modelling of CYP102A1 monomer. Relative orientations of the poses were adjusted for comparison purpose using orientation of the P450 domain in P14779 structure as a reference. The relative orientation of the FAD domain compared to the P450 and FMN domains that formed a complex appeared highly variable in the presented AF2A predicted poses. P450d, FMNd, and FADd are colored red, yellow, and blue, respectively.

The competitive modelling approach was designed considering that the CYP102A1 FMNd must form alternate electron transfer complexes with the FADd and the P450d to support catalytic cycles, but that a direct electron transfer from FADd to P450d is not possible. In the dimeric structure formation of intra- or inter-chain complexes critical for catalysis can be considered in addition to structural interchain contacts between the heme, FMN and FAD containing domains. Considering that AF2A modelling generally results in a unique predicted conformation for binary complexes, a tripartite modelling strategy was designed to favor the generation of alternate structures by competition. The set of the different modelling assays we carried out for this work is summarized in Fig. 1a.

#### Full-length monomer modelling

A structural model for the full-length monomer of CYP102A1 was previously released in the AlphaFold Protein Structure Database (Entry P14779). This model was compared to several alternate outputs of our AF2A predictions (Fig. 1b–c). Predicted structures for the FADd, FMNd and P450d taken individually were almost identical (RMSDs < 1.5 Å) between the newly generated models, the previously released structure and the available crystallographic references (PDB: P450d, 4kew; P450d-FMNd, 1bvy; FADd, 4dqk). Particularly, the RMSDs between the best AF2A models and crystal structures for the P450d-FMNd complex (PDB 1bvy) or for the FADd (PDB 4dqk) were respectively 0.88 Å and 1.03 Å. In all considered models, distances and orientations between the two redox cofactors (heme and FMN) are compatible with efficient electron transfers. In contrast, relative orientations of FADd with respect to the FMNd-P450d complex appeared extremely variable within the limits permitted by geometry of the polypeptide chain linking FMNd to FADd. This observation is consistent with the high flexibility of the FMNd-FADd hinge region that makes relative orientation of linked domains poorly defined in the absence of complex formation. This illustrated that AF2A was unable in this case to predict the alternate conformation in which the FMNd forms an alternate complex with FADd, leading to a closed conformation of the reductase domain. However, modelling of this monomeric structure was not representative of the experimental situation in which the catalytically active form of CYP102A1 is known to be dimeric^18^.

#### Dimer modelling of P450d-FMNd part

**Figure 2:**
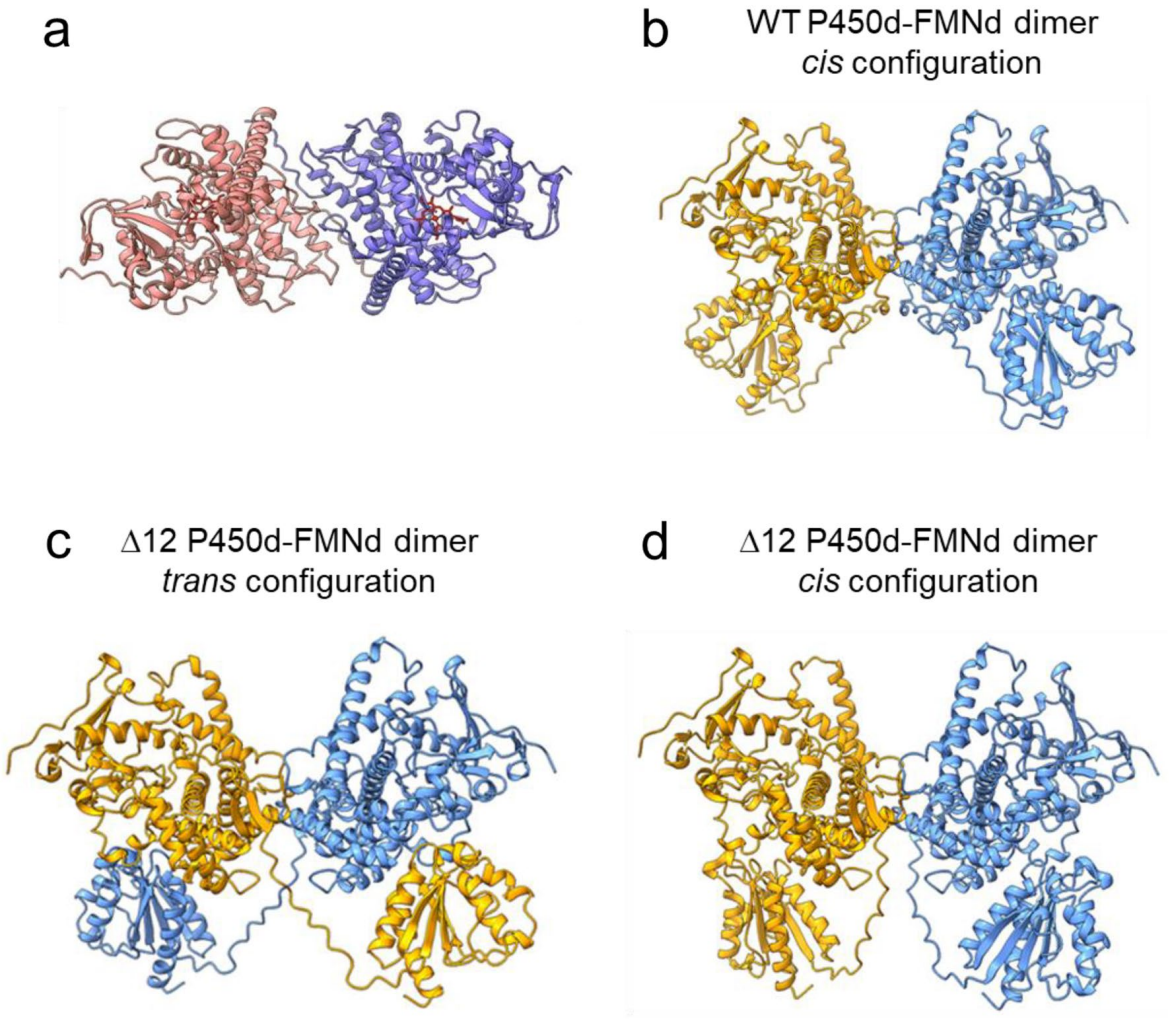
AF2A modelling of CYP102A1 P450d-FMNd parts. (**a**), Predicted AF2A structure for the P450d dimer (CYP102A1 residues 2–458). The two heme cofactors are in red. (**b**), Predicted AF2A structure for the P450d-FMNd dimer (CYP102A1 residues 1–631). (**c–d**), Predicted AF2A structures for the corresponding variant featuring a 12-residue (^457^GGIPSPSTEQSA^468^) truncated P450d-FMNd linker. Both crossed (*trans*) and non-crossed (*cis*) predicted configurations are shown. Domains belonging to the same polypeptide chain share the same color.

Possible dimerization of the isolated heme-containing domains was first evaluated. AF2A modelling systematically generated a unique structural organization (in 10 out of 10 runs) in which a pair of isolated P450 domains formed a well-defined binary complex (Fig. 2a). Presence of two P450d per asymmetric units was also observed in crystal structures PDB 6h1s and 4kew, but with a markedly different geometry of the interfaces between experimental structures that also differ from the ones in AF2A and CryoEM models. Predicted P450d interfaces during repeated AF2A modeling were in contrast almost identical. Concerning P450d taken individually, AF2A predicted and crystal structures PDB 6h1s and 4kew were highly similar with a RMSD of 0.77 Å (413 Cα/453) and of 0.74 Å (381 Cα/400), respectively. The free energy for the formation of the P450d-P450d interface was estimated to be − 9.3 kcal/mol for the AF2A model (− 14.7 kcal/mol following side chain structure refinement), compared to − 5.2 and − 7.0 kcal/mol for the associations seen in crystal structures. Experimentally, dimerization of isolated CYP102A1 heme domains was not observed in solution based on size exclusion chromatography^18^. In contrast, sedimentation velocity experiments on partially proteolyzed CYP102A1 evidenced an unusual stoichiometry that was interpreted as the binding of a full-length CYP102A1 monomer to an isolated proteolyzed P450d^35^. Considering this observation, the structural organization of the P450d complexed to FMNd in the dimers was also analysed (Fig. 2b). Complexation with FMNd did not influence the previously predicted interface between the heme containing domains. The two FMNd formed symmetrical complexes suitable for electron transfers with the P450d proximal faces, opposite to the P450d-P450d interface. Geometry of P450d-FMNd complexes appeared highly similar to the one previously described for CYP102A1 monomer and observed in the crystal structure PDB 1bvy (Supplementary Figure S1a).

Considering now the flexible linker between P450d and FMNd, a *cis*- or a *trans*-configuration for the linker can be considered in the dimer, constituting mutually exclusive models. Repeated AFA2 modelling of a pair of wild-type P450d-FMNd sequences always placed the P450 and FMN domains in a *cis*-conformation (Fig. 2b). In contrast, modelling of a pair of variant sequences featuring a 12-residue (Δ^457^GGIPSPSTEQSA^468^) shortened P450d-FMNd linker stochastically generated both the *cis* and the *trans* configurations in similar proportions (Fig. 2c–d). This truncated variant was designed and used in a previous cryo-EM study to significantly improve resolution during imaging of the CYP102A1 dimer, while causing CYP102A1 inactivation^24^. Consistently, AF2A predictions illustrated a binding angle of FMNd to P450d tilted by about 38 degrees when the P450d-FMNd linker is shortened (Supplementary Fig. S1b–d). Such tilting is inconsistent with crystal structure of the monomeric complex, potentially impairing electron transfer, and could explain the experimentally observed inactivation. In this case, alternate AF2A predictions could result from the fact that FMNd tilting is reducing the stretch of the shortened polypeptide chain. The difference of frequencies of the AF2A-modelled *cis-* and *trans-*configurations may thus reflect the higher geometrical constraints on the *cis*- compared to the *trans*-configuration and suggests that AF2A arbitrations can result from differential geometric constraints on alternate structures. While no direct experimental evidence has been published allowing to quantify contributions of *cis*- and *trans*-conformations in solution, indirect biochemical evidences involving heterodimers associating site-directed mutants^18,19^ suggested that both conformations are involved in catalysis. This point was developed in the extended BioRxiv preprint version of this paper.

#### Structure of the dimer of FMNd-FADd part evidences an unusual crossed configuration

**Figure 3:**
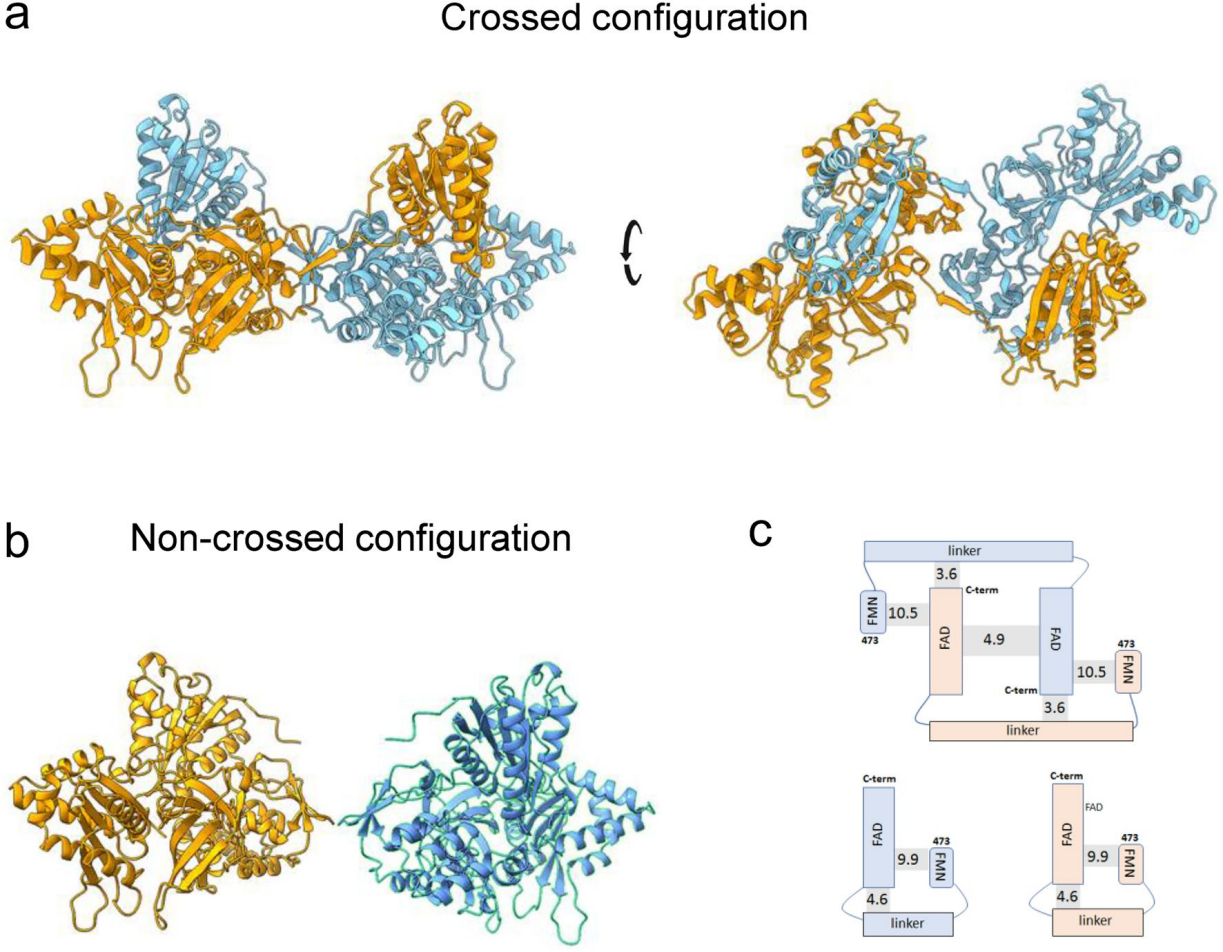
AF2A modelling of CYP102A1 FMNd-FADd parts. (**a**), Predicted structure in two orientations of a pair of CYP102A1 FMNd-FADd parts (residues 473–1049) forming a dimer in a crossed configuration. (**b**), Corresponding monomeric structures alternatively predicted. Orientation of the FADd was set identical to that in (**a**). (**c**), Free energies of interaction between the different sub-structures in the crossed dimer (top of panel **c**) and monomers (bottom of panel **c**) were calculated using PRODIGY. Number refers to amino acid positions in CYP102A1 sequences. Values on shaded area joining sub-structures are corresponding ΔG contributions to interchain interactions (for dimer) or intrachain FADd-FMNd interaction (for monomer).

The CYP102A1 part comprising only FADd and FMNd is the equivalent of the strictly monomeric CPRs supporting activities of P450 enzymes in eukaryotes. Consistently, AF2A modelling of pair of CPR chains from various eukaryotic origins (animals, plants, yeast) was attempted and always generated monomeric structures. Surprisingly enough, AF2A modelling of pair of CYP102A1 FADd-FMNd parts mostly generated (in about 80% of attempts) a new dimeric structure in which the FMNd of one monomer forms a complex with the FADd of the other monomer and reciprocally (Fig. 3a). Such a crossed geometry was never previously described for any diflavin reductases. A minor fraction of AF2A modelling runs generated the alternate classical structure (Fig. 3b) similar to that of eukaryotic CPRs in which FADd and FMNd form a closed intrachain complex. Apart from the geometry of the polypeptide chain region linking FADd to FMNd, the geometries of modelled complexes were highly similar in the crossed and noncrossed structures, particularly leading to identical relative geometries of FMN and FAD cofactors consistent with electron transfers capabilities as seen in known crystal structures of eukaryotic CPRs^36^.

In the crossed model, dimer formation resulted from the three different types of interactions (Fig. 3c). Binding free energy (ΔG) resulting from the interchain complex formation between one FMNd and one FADd was estimated to be − 10.5 kcal/mol, thus contributing for − 21 kcal/mol to the dimer stability considering the symmetry. The − 10.5 kcal/mol value was very similar to the value of − 9.9 kcal/mol estimated for the corresponding intrachain complex in the monomeric CPR structure. In the dimer, the interaction between the FADd of one monomer and the linker region connecting FMNd to FADd of the other monomer additionally was estimated to contribute for 2 x − 3.6 kcal/mol (due to symmetry), and the interface between the two FADd for − 4.9 kcal/mol. The total binding energy between the two reductase parts in the crossed dimer was thus in the range of − 33 kcal/mol compared to − 5 kcal/mol for the sole contributing FADd-FADd interface in the cryo-EM deduced model. Such − 5 kcal/mol value would appear particularly low in regard to the experimental dissociation constant in the nM range for CYP102A1 dimer^18^.

### Structural assembly of the full-length CYP102A1 dimer into alternate geometries is consistent with catalysis

**Figure 4:**
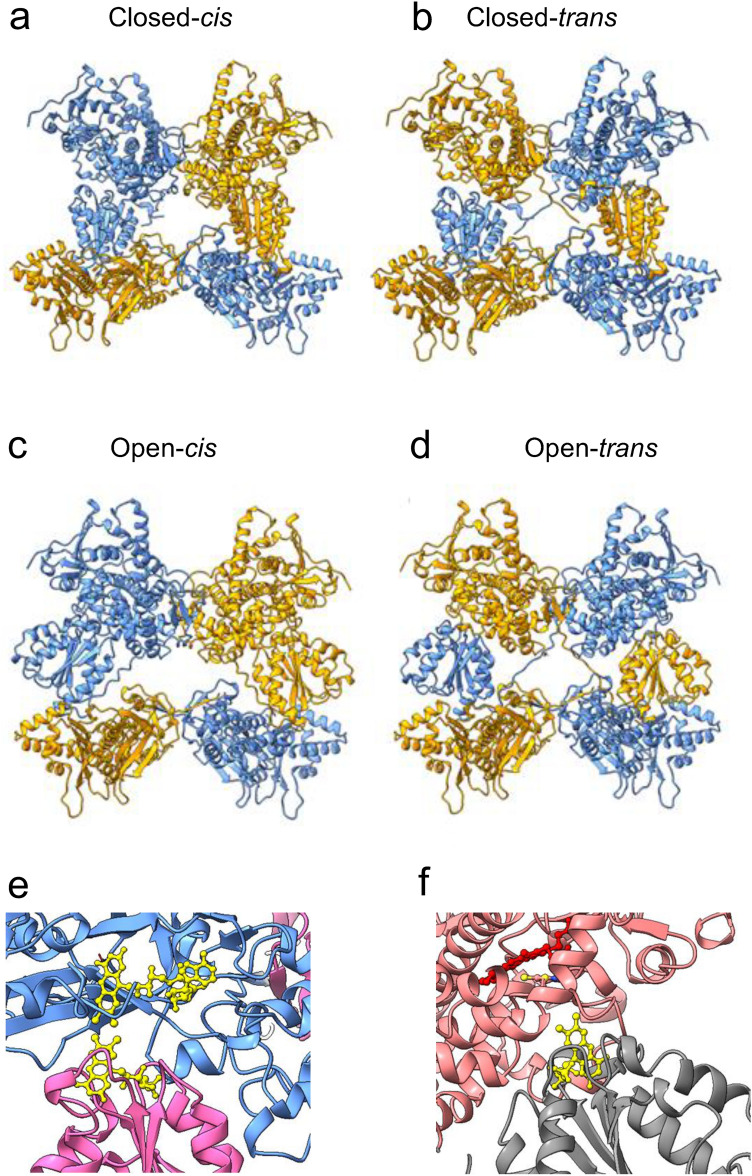
Predicted assembled structures for the open and closed conformations of a pair of full length CY102A1. (**a–b**), In the closed conformations, FMNd forms electron transfer competent complex with FADd. (**c–d**), In the open conformations, FMNd forms electron transfer competent complexes with P450d; *cis* and *trans* refers, respectively, to non-crossed and crossed topologies of the P450d-FMNd linker. In the open conformation, the linker between FMNd and FADd is not represented (see “Methods” section). The two AF2A-predicted chains in the dimer are colored differently for the sake of clarity. (**e–f**), Geometries of cofactors in the closed (FMN and FAD in **e**) and open conformations (FMN and heme in **f**). Flavins are colored yellow and heme colored red.

The previously described models constitute mutually exclusive associations of FMNd with either P450d or FADd. Consequently, full CYP102A1 models cannot be directly assembled from these non-complementary parts. Interconversion between the two catalytic structures requires dissociation of the FMNd from its interface with FADd to form its counterpart with P450d. However, mechanism in the dimer can be asynchronous for the two FMNd and is not expected to require major conformational changes at the FADd-FADd interface. We hypothesized that geometry of this interface was mostly conserved during conformation changes required for turnovers. The same consideration applies to the P450d-FMNd complex as its dissociation is not expected to interfere with the stability of the P450d-P450d interface. Consequently, a first global model of the CYP102A1 dimer was assembled with the reductase domains in a closed configuration from both the P450d dimer and the FAD-FMNd dimer models (Fig. 4a–b). In this model distance and orientation of two flavin cofactors are similar to those found in CPRs (Fig. 4e). A second model with the reductase domains in an open configuration was in contrast assembled from the P450d-FMNd and the FADd-FADd dimer models (Fig. 4c–d). This last model was deduced from the FADd-FMNd crossed dimer structure by erasing duplicated FMNd before assembly without changing FADd atoms coordinates. In this model the FMN and heme cofactors shares a common geometry with previously modelled electron transfers complexes (Fig. 4f). Both structures being homodimers, the partial structures must share a common symmetry axis. Final models were thus assembled by adjusting the distance between barycenter and the rotation angle of partial models along this common symmetry axis. The distance was adjusted to the shorter value not creating structural clashes. Concerning the angle, two approaches were considered: one ab initio by assuming that the dihedral angle between the P450d and FADd dimers was similar in the open and closed conformations thus minimising required conformational changes, the other by optimizing the embedding quality of models into the previously reported cryo-EM density maps^24^. Crossed (*cis*) and non-crossed (*trans*) FMNd-P450d geometries were considered during reconstructions generating two alternate conformations for each of the open and closed structures.

**Figure 5:**
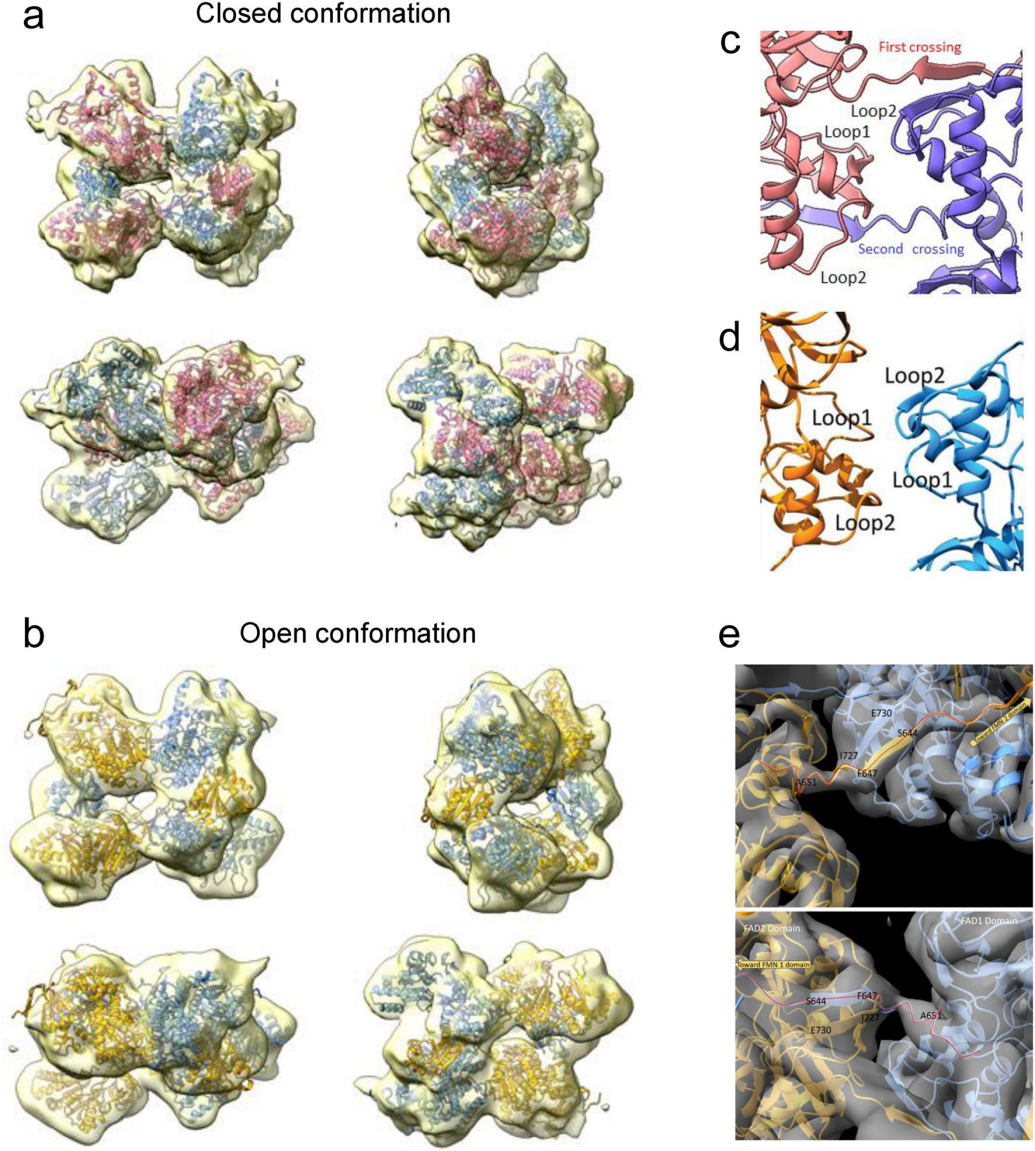
Comparisons of AF2A-predicted structures and experimental cryo-EM envelope of full-length CYP102A1 dimer. (**a–b**), The AF2A models (*trans* P450d-FMNd geometry) for the Δ12 CYP102A1 variant in the closed (in **a**) and open (in **b**) conformations were embedded into cryo-EM EMD-20785 density map as described in “Methods” Section. The same color code was used for domains belonging to the same polypeptide chain. Experimental cryo-EM map is colored light yellow. Similar orientations were selected to help visualize the open and closed structures in the four presented orientations. (**c–d**), Comparison of the FADd-FADd interface in AF2A (top) and reported cryo-EM (bottom) models. Loop1 and loop2 correspond to the 8-residue long (residues 647 to 655) chain that crosses the FADd-FADd interface in the dimer. This loop exhibits an extended configuration in the dimer and crosses the interface (in **c**), while it turns back to the FADd of the same monomer in the reported cryo-EM model (in **d**). (**e**), Closer views of the FADd-FADd interface using the high resolution EMD-21100 density map (grey color) as a reference and the AF2A-modeled chains for the FMNd-FADd part in the closed conformation. The two AF2A-predicted chains in the dimer are colored differently for the sake of clarity. The thin red line in (**e**) highlights the course of the two crossing chains.

The resulting open and closed models were found highly consistent with reported EM density maps as illustrated under different view angles (Fig. 5a–b). Simulated electron densities from models well matched with cryo-EM maps at the levels of P450d and FADd models with a similar RMSD of 2.4 Å. However, in the closed conformation, part of the EM density map enveloping the FMNd appeared significantly larger than the maximum volume that can be occupied (Fig. 5a). Interestingly, predicted electronic densities associated with the open conformation filled complementary volumes at FMNd level (Fig. 5b and Supplementary Fig. S2b). This suggested that the enlarged experimental EM map could result from the averaging of densities belonging to the two alternate conformations. The other minor visible misfit occurring around the N-termini of the two monomers resulted from the absence in the AF2A models of the N-terminal His-tag extension present in experimental imaging.

Comparison of the open and closed models illustrated that the two conformations are compatible with a similar positioning of the dimeric P450d and FADd parts within a same EM density envelope (with some limitations for the *trans* open conformation, see “Methods” section). No direct contact exists between P450d and FADd, but their relative orientations are constrained by the maximal extensions of polypeptide chains associating P450d and FADd to FMNd. It is important to note that while our modelling approach involved linker cleavages to generate partial models, reestablishing connectivity on the fully assembled structures was always found geometrically feasible without introducing major structural constraints.

FMNd rotation required during catalysis for the conversion between alternate complexes could be sterically hindered in the absence of some structure relaxation. This could be performed for example by a transient motion of the two contacting FADd into CYP102A1 dimer, which would be greatly facilitated by the dissociation of one of the interchain FADd-FMNd complex following some remodelling of the weak interactions (− 4.9 kcal/mol) maintaining the FADd-FADd interface. The resulting partially opened structures could be consistent with alternate images reported in cryo-EM data^24^. An asymmetric (closed-open) hybrid conformation of the full-length CYP102A1 dimer was built and is illustrated in Supplementary Fig. S2a–b. This model illustrates links between alternate geometries and the two subsequent electron transfers involved in catalytic cycle.

The P450d-P450d interface in the modelled full-length dimers was compared in more details to reported cryo-EM data. As illustrated, AF2A predicted and cryo-EM imaged interfaces were highly similar, including for the nature and position of contacting amino acid residues (Supplementary Fig. S3a–b). This suggested that formation of this interface was autonomous and independent of structural interactions in other parts of CYP102A1 structure and could constitute alone a determining factor for the dimerization of full-length protein. Considering now the FADd-FADd interface, AF2A and cryo-EM models significantly differed at level of the connectivity of the polypeptide chain linking FMNd and FADd. In the AF2A structure, the FMNd-FADd linker, which encompasses the 12-residue hinge segment (residues 632–643) and also the N-terminal loop of FADd (residues 644–654) is crossing the FADd-FADd interface, thus permitting formation of an interchain FADd-FMNd complex (Fig. 5c). In contrast, no chain crossing the interface is present in the cryo-EM model (Fig. 5d) resulting into formation of an intrachain FMNd-FADd complex. To solve the contradiction, the high resolution cryo-EM density map EMD-21100 was reexamined. Taken individually, the folds for the FMNd and the FADd were highly similar between the AF2A models and to the corresponding crystal structures (Supplementary Table S2). The elements of the two FMNd-FADd linkers crossing the FADd-FADd interface in the AF2A models can be unambiguously visualized, and AF2A-predicted structure well fitted within the cryo-EM map (Fig. 5e). Structures of the FADd-FADd interface deduced from AF2A and cryo-EM models also involve the same orientations and contacting residues. Results supported the prediction of a novel alternate structure for diflavin reductase enzymes in which the FMN domain of one monomer is associated to the FAD domain of the other monomer, contrasting with the monomeric conformation of eukaryotic CPRs. The C-terminal extremity (Loop 1) of the β-sheet extension of FMNd linker can adopt two alternate conformations: one forming an intra-domain loop with the FADd of the same monomer, or the other forming an inter-domain loop with the FADd of the other monomer (Supplementary Fig. S3c).

The chain connectivity at level of the P450d-FMNd linker was also reexamined. Reported cryo-EM data interpretations were based on a CYP102A1 variant in which this linker was shortened by 12-residues to improve the insufficient density maps resolution achieved with wild type enzyme^24^. A *trans* geometry was favored in the cryo-EM report based on modelling of the P450d-FMNd linker electron density as a polyalanine sequence. In contrast, *cis* and *trans* geometries (Fig. 4) appeared equally predicted by AF2A runs using the truncated sequence when only *cis* geometry was predicted for the native sequence. A clear-cut conclusion thus remained difficult to reach. However, the 12-residue deletion was detrimental to activity and could have experimentally favored formation of a *trans* conformation not necessarily representative of the native structure.

### Structural features favoring dimerization in single chain bacterial monooxygenases

Dimerization of the P450 and of the reductase domains are structurally independent events in the absence of direct P450d-FADd interaction in the full-length enzyme. The NCBI database was PBLAST-searched for sequences similar to the CYP102A1 reductase domain (excluding its P450 domain). Surprisingly, hits featuring more that 50% identity were all natural fusions with a P450. The hits were filtered to remove duplicates or too similar sequences and were clustered to finally retain five typical sequences belonging to different microorganisms exhibiting variable identity with the target (54 to 95%). Their fused P450 domains exhibited 64 to 98% amino acid identity with that of CYP102A1 (Supplementary Table S3). AF2A modelling of these sequences predicted that all of them share the same structural organization as CYP102A1, with an independent dimerization of the P450 domains and the formation of a reductase dimer stabilised by a crossed FADd-FMNd association. The calculated binding free energies of interfaces were found very similar between them and with those of CYP102A1. Interestingly, the P450d-P450d interfaces in the five selected sequences encompass 18 highly conserved residues forming contacting pairs at the interface in more than 50% of AF2A modelling attempts (Supplementary Fig. S3b). In contrast, a monomeric structure was AF2A predicted for the three other *Priestia megaterium* P450 sequences not fused to a reductase (WP_029321191.1, WP_013058569.1, and WP_053488633.1). Similarly, the unique *P. megaterium* sequence (WP_057244461.1) homologous to CYP102A1 reductase domain but not fused to a P450 was predicted to fold as a monomer.

**Figure 6:**
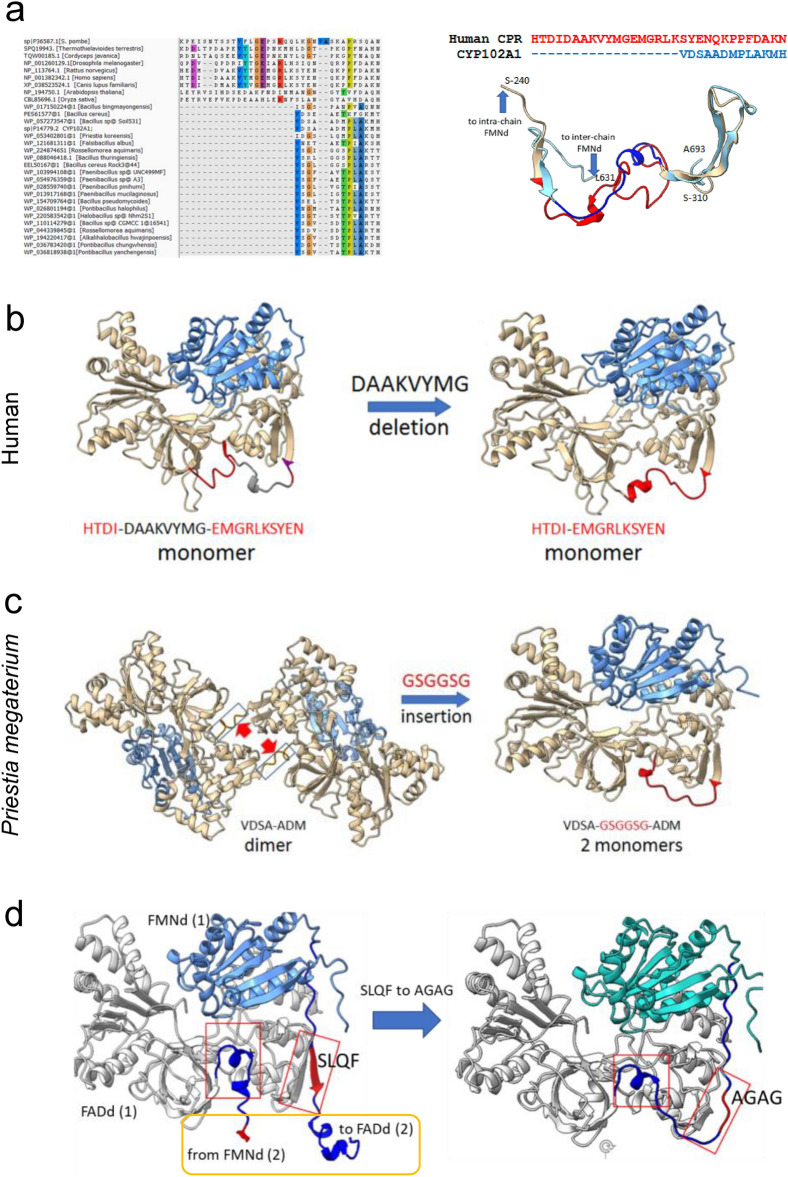
Structure determinants for dimerization identified by in silico mutations of human CPR and bacterial CYP102A1. (**a**), Sequence (left) and structural (right) alignments for the FMNd-FADd interdomain linker region of bacterial and eukaryotic diflavin reductase domains. Structure for human CPR (PDB 3qe2) from Ser240 to Ser310 (colored in wheat) with His250 to Asn280 part is red. The corresponding modeled structure for CYP102A1 is in pale blue with the Val648-His660 in dark blue. The common β-sheet used as alignment limits are visible on the right and left part of the red and deep blue parts. (**b**), Structural consequence of the ^254^DAAKVYMG^261^ sequence deletion (in grey) in the interdomain linker region (in red) of the human CPR. (**c**), Predicted structural consequence of a GSGGSG sequence insertion (in red) in the same interdomain linker region (^648^V-M^654^, red arrows) of dimeric CYP102A1. The insertion converted the dimer into two monomers featuring similar structures with human CPR (**b**, **c** right). (**d**), predicted structural consequences of the ^644^SLQ F^647^ by AGAG (in red) sequence substitution in the sequence segments (in blue) crossing the FADd-FADd interface in dimeric CYP102A1. The dimeric structure is converted into two monomers.

To evaluate factors controlling the dimerization, sequences of discussed bacterial fusions were compared to eukaryotic CPR ones. The length of the FMNd-FADd linker (hinge + N-terminal loop of the FADd) was found to be systematically shorter by about 20 amino acids in CYP102A1 and related enzymes compared to monomeric eukaryotic CPRs (Fig. 6a). Based on this, a CYP102A1 variant was designed in which this linker region is extended by 6 amino acids, forming a new highly flexible sequence segment, transforming the wild-type ^648^VDSAADM^654^ sequence into VDSA**GSGGSG**ADM. Reciprocally, the corresponding region in the human CPR was deleted, transforming the wild-type ^250^HTDI**DAAKVYMG**EMGRLK^267^ in the shorter HTDIEMGRLK sequence. Figure 6b illustrates that the eight residues deletion in human CPR did not significantly modify the predicted CPR folding except for some structuration of the shortened loop with a supplemental short α-helix. No dimerization was predicted upon repeated modelling (up to 10 runs). In sharp contrast, a major conformational change was predicted when the linker of the bacterial enzyme was extended by six residues. The insertion converted the reductase domain from a dimeric to a strictly monomeric form similar to that of monomeric eukaryotic CPRs (Fig. 6c). This result clearly suggested that the shortened FMNd-FADd linker systematically found in dimeric bacterial enzymes was critical for dimer formation.

The absence of reciprocity upon linker extension in the monomeric CPR suggested that the shortened linker of bacterial enzymes more likely favor dimerization by destabilising specifically the intrachain complex. Particularly, dimer formation also requires that a compatible FADd-FADd interface can be formed. The role of CYP102A1 amino acids 644–647 that forms a small β-sheet with a complementary β-strand from the FADd was also questioned. This motif constitutes in CYP102A1 the entry point of the interdomain linker at the FADd-FADd interface immediately upstream of a characteristic turn present in all eukaryotic CPRs but systematically absent in CYP102A1 (Figs. 5c and 6a). In eukaryotic CPRs, this loop (Loop 1) goes back into the FADd, whereas the presence of the β-strand in CYP102A1 directs the chain toward the other monomer. The native ^644^SLQF^647^ sequence of this β-strand was substituted in silico by the ^644^AGAG^647^ sequence in order to try to destabilise this fold. AF2A modelling of the variant has shown prediction of two alternate structures with a similar frequency (Fig. 6d). One was monomeric (right) with an intramolecular FADd-FMNd closed configuration, while the other was dimeric (left) and similar to the native enzyme. The FMNd-FADd linker adopted a different orientation in the two structures, the small β-sheet existing only in the native crossed complex. However, this conclusion must be mitigated considering that relative proportions of dimeric and monomeric structures predicted appeared variable when testing different sequence substitutions.

Finally, the role in CYP102A1 dimerization of the structural complementarity at the FADd-FADd interface was evaluated. For that, a chimeric human-bacterial sequence was designed in which the sequences of the FMN domain and the FMNd-FADd interface were those of human enzyme, while the sequence of the FADd-FADd interface and the linker region were those of bacterial reductase domain (Supplementary Table 4). While the structure of the native human enzyme was always predicted to be monomeric, in 2 cases out of 10 (20%) AF2A successfully predicted that the chimeric enzyme indeed adopts the expected dimeric structure with a crossed configuration typical of CYP102A1 (Supplementary Fig. S4). This score of 20% was significantly lower than the success rate (80%) for dimer prediction with the wild-type CYP102A1 sequence, but was in the same range than the 20% value obtained for the dimeric geometry of the PES61577 sequence from *Bacillus cereus* that shares 68% amino acid identity with CYP102A1. Predicted formation of the dimeric chimeric structure in which only the FADd-FADd interface region is of bacterial nature, appears of particular interest for the engineering of eukaryotic monooxygenases offering a route to build synthetic dimeric eukaryotic P450-CPR fusions mimicking the structure of highly efficient bacterial enzymes.

## Conclusions

The present work illustrates that AF2A shares with the more finalized versions, noticeably AlphaFold-multimer, the capability to accurately model individual domains of modular proteins as well as the structure of potential intrachain or interchain complexes. However, and as previously reported, fold switching or alternate domain interactions are generally hardly predicted^8,9^, limiting its potential to address large-scale structural dynamics critical in major biochemical functions^37,38^. Using CYP102A1 as a test case, we devised a bypass to these limitations through a ‘*modelling by competition*’ strategy. To do so, enabling factors were: (i) the decomposition of the global model into non-competitive sub-models allowing prediction of all possible intrachain and interchain complexes; (ii) the software itself by challenging it through a set of competitive modelling in which alternate and mutually exclusive complexes can be formed with relative frequencies of alternate predictions resulting from conflictual geometric arbitrations. Underlying algorithmic mechanisms and basis of their physical relevance are unclear to us, but the results we present evidenced that these undocumented mechanisms are logically consistent and sensitive enough to predict the effect of small sequence deletions, insertions or mutations in large structures. This gives the AF2A tool a strong potential for rational design, as suggested by the presented proof-of-concepts illustrating the potentiality to rationally drive quaternary structure changes.

Concerning more specifically the bacterial monooxygenase CYP102A1, our results suggested that formation of the unusual crossed reductase structure constitutes a new fold for diflavin reductases and plays a critical role for the particular high efficiency of the enzyme, independently of the dimerization of its P450 domains. Independent dimerizations of the FAD and heme-containing domains generate a stator in which the two FMN domains act as an oscillating rotor to establish a highly performing electron transfer chain. Several complementary factors were identified that affect the potential conformational equilibrium between a monomeric structure enabling intrachain interflavin electron transfer and a crossed dimer enabling interchain electron transfers. Presented results trace a route for a reverse engineering of eukaryotic monooxygenase systems based on the unusual structure of their bacterial fusion counterparts.

## Supplementary Information

Supplementary Information 1.

## Data Availability

Atomic coordinates of the different AF2A-predicted CYP102A1 models (both full-length and partial) are available by emailing the authors: P.U. at urban@insa-toulouse.fr or D.P. at dpompon@insa-toulouse.fr. A preliminary version of this paper presenting complementary biochemical information was published on the BioRxiv: https://www.biorxiv.org/content/10.1101/2022.03.21.485149v1.

## Abbreviations

AF2A: AlphaFold2_advanced
cryo-EM: Cryo-electron microscopy
CMA: Competitive modelling approach
FADd: CYP102A1 FAD-binding domain
FMNd: CYP102A1 FMN-binding domain
P450d: CYP102A1 heme-binding domain
P450: Cytochrome P450
