# Supplementary material for: Confrontation of AlphaFold models with experimental structures enlightens conformational dynamics supporting CYP102A1 functions
Source: Sci Rep. 2022 Sep 25;12:15982. doi: 10.1038/s41598-022-20390-6 (PMC9510131; doi:10.1038/s41598-022-20390-6)
Supplement: Supplementary file 1 — Supplementary Information 1. [file 41598_2022_20390_MOESM1_ESM.docx]

**Supplementary Material**

**Confrontation of AlphaFold models with experimental structures enlightens conformational dynamics supporting CYP102A1 functions**

Philippe Urban & Denis Pompon

**Supplementary Figure S1. Comparison of predicted and crystallographic structures for CYP102A1 P450d-FMNd complexes**. **a**, Overlay of crystal structure 1bvy (coloured in sand) with AF2A model (in pale blue) following RMSD minimization restricted to P450 domains. **b**, AF2A predicted structure in the *trans*-conformation. P450d (in sand) and FMNd (in red) formed symmetrical complexes opposed to the P450d-P450d interface. The crossed linker region is depicted in blue. **c**, Overlay of the RMSD-minimized predicted (in sand) and crystallographic structures (in red) of the FMN domain. **d**, Close view of the P450d-FMNd interfaces: only half (with the symmetry axis represented by the dashed line) of the symmetrical AF2A structure (in pale blue) and the part of the crystallographic structure (P450 domain coloured in sand and FMN domain in red) involving the P450d-FMNd complex shown.

**Supplementary Figure S2. Predicted asymmetric model combining open and closed conformations of the reductase domain. a,** Composite view for an assembled model of the full length CYP102A1 dimer in which the left part is on the closed (its FMN domain is coloured red) and the right part in the opened conformation with the FAD domain coloured red and the P450 and FMN domains coloured green. **b**, Embedding of the hybrid model into the experimental EMD-20786 electron density map of CYP102A1 A82F mutant. **c**, Surface envelop representations of the hybrid model in two opposite orientations permitting either the FADd to FMNd or the FMNd to P450d electron transfers (yellow arrows). The two polypeptide chains are respectively colored in blue and rose with the FMN domain of one monomer being highlighted in purple.

**Supplementary Figure S3. Comparison of modeled and experimental structures for the P450d-P450d interfaces and for the monomeric and dimeric diflavin reductase parts. a,** Comparison in similar orientations of the P450d-P450d interface in the AF2A dimeric model (left) and in the published cryo-EM structure (right). **b**, View of interacting side chains (at < 4 Å) at P450d-P450d interface. Selected side chains are in grey color with their nitrogen and oxygen atoms in blue and red. **c**, Overlay of AF2A-predicted structures for the FMNd-FADd dimer (chains in purple and pink) with corresponding monomer structure (in green). Loop1 and 2 exhibit extended configuration in the dimer crossing the interface while turning back to the FADd of the same chain in the monomers.

**Supplementary Figure S4. Conversion of monomeric CPR to a dimeric structure in a synthetic mosaic diflavin reductase. a,** Predicted dimeric structure of CYP102A1 reductase domain association (given here as a reference) with one chain coloured in cyan and the other chain coloured in magenta. **b**, Predicted dimeric structure (two orientations) for the synthetic chimeric sequence (named SYN001). Pale blue (FADd) and magenta (FMNd) parts are sequences of human origin and yellow parts (FADd) of bacterial origin. **c**, Alternate structural prediction of the monomer (in two orientations) using the same color code as in panel **b**.

**Supplementary Table S1.** PRODIGY calculated binding ΔG for the P450d-P450d interface of unrelaxed AlphaFold, Rosetta-minimised AlphaFold and crystallographic models.

| **Model type** | **pTM rank** | **Complex geometry** | **P450d-P450d interface free energy (kcal/mol)** |
| --- | --- | --- | --- |
| AF2A | 1 | 1 | -9.3 |
| AF2A | 2 | 1 | -10.0 |
| AF2A | 3 | 1 | -8.7 |
| AF2A | 4 | 1 | -9.2 |
| AF2A | 5 | 1 | -9.6 |
| AF2A + Rosetta | - | 1 | -14.7 |
| PDB 4kew | - | 2 | -7.0 |
| PDB 6h1s | - | 3 | -5.2 |

Comparison of free energies for interface formations in best ranked unrelaxed AlphaFold models and the same models after relaxation with Rosetta.

| **Modelled dimer** | **Domain interface** | **Best AF2A model**  **kcal/mol** | **Rosetta relaxed**  **kcal/mol** |
| --- | --- | --- | --- |
| P450d dimer | P450d-P450d | -9.3 | -14.7 |
| P450d-FMNd dimer | P450d-FMNd | -8.0 | -10.1 |
| FMNd-FADd dimer | FADd-FADd | -12.1 | -13.6 |
| FMNd-FADd dimer | FADd-FMNd | -10.7 | -11.8 |

**Supplementary Table S2. RMSD between AF2A predictions and crystal structures for different CYP102A1 subparts.** Both P450d-FMNd and FMNd-FADd linker regions were excluded from calculation due to their flexibility. CYP102A1 heme domain from PDB structures 4kew and 6h1s were considered for RMSD minimization from the residue Met6 (MPQP sequence) to Lys453 (KIPL sequence). FMNd from PDB structure 1bvy was considered from the residue Asn480 (NTPLL sequence) to Tyr628 (YFNL sequence). FADd from PDB structure 4dqk was considered from His660 (in HGAF sequence) to Lys1044 (in KDVWA sequence at the C-terminus). Numbering refers to full length CYP102A1 sequence. AF2A structures used for comparisons were extracted from model of the full-length monomer for P450d and FMNd comparisons and from the closed FMNd-FADd structure for FADd comparison.

| **Enzymes** | **Crystal Structures** | |
| --- | --- | --- |
| P450 domain | PDB 6hs1 | PDB 4kew |
| AlphaFold model | 1.14 Å | 1.18 Å |
| PDB 6hs1 | - | 0.75 Å |
| Reductase domain | PDB 1bvy | PDB 4dqk |
| AlphaFold FAD domain | - | 1.03 Å |
| AlphaFold FMN domain | 0.88 Å | - |

**Supplementary Table S3.** **Similarity, tendency to dimerize and domain interactions in CYP102A1 related enzymes**. Predicted tendency to dimerize was evaluated as the fraction of dimeric structures predicted by AF2A for pair of P450d, and FMNd-FADd parts of the full-length enzymes. The surface of the buried interface in dimers and the binding free energy of the corresponding complex association were calculated with PRODIGY. Sequence id referred to GenBank entries.

| **Sequences** | **CYP102A1**  **ADB P14779** | **WP**  **057273547** | **WP 053402801** | **PES 61577** | **WP 044339845** | **COF 77949** | **WP 003242884** |
| --- | --- | --- | --- | --- | --- | --- | --- |
| Origin | *Priestia megaterium* | *Bacillus sp* | *Priestia koreensis* | *Bacillus cereus* | *Rossellomorea aquimaris* | *Sterptococcus pneumoniae* | *Bacillus subtilis* |
| P450 domain identity | 100 % | 98 % | 87 % | 79 % | 64 % | ND | 64 % |
| Reductase domain identity | 100 % | 95% | 73 % | 68 % | 57 % | 56 % | 54 % |
| P450 domain predicted as a dimer | 5/5 | 5/5 | 5/5 | 3/3 | 5/5 | ND | 4/5 |
| Reductase domain predicted as a dimer | 4/5 | 3/5 | 3/5 | 1/5 | 4/5 | 5/5 | 4/5 |
| P450d-P450d interface  surface | 2473 Å^2^ | 2713 Å^2^ | 2536 Å^2^ | 2309 Å^2^ | 2377 Å^2^ | ND | 2717 Å^2^ |
| P450d domain  ΔG binding (kcal/mol) | -9.3 | -8.7 | -10.0 | -11.5 | -10.5 | ND | -11.3 |
| Reductase domain ΔG binding (kcal/mol) | -19.8 | -20.9 | -20.0 | -20.9 | -22.8 | -19.8 | -17.6 |

**Supplementary Table S4. Chimeric human-bacterial enzyme sequence.** This sequence that we have named SYN001, begins with the residue 75 of human CPR (Lys75). The missing sequence (1-74) corresponds to the original membrane anchoring sequence of human CPR.

KMKKTGRNIIVFYGSQTGTAEEFANRLSKDAHRYGMRGMSADPEEYDLADLSSLPEIDNALVVFCMATYGEGDPTDNAQDFYDWLQETDVDLSGVKFAVFGLGNKTYEHFNAMGKYVDKRLEQLGAQRIFELGLGDDDGNLEEDFITWREQFWPAVCEHFGVEATGEEDNKSTLSLQFVDSANQKPPFDAKNPFLAAVTTNRKLNQGTERHLMHLELDISDSKIRYESGDHVGVIPRNYEGIVNRVTARLGLDASQQIRLEAEEEKLAHLPLAKTVSVEELLQYVELQDPVTRTQLRAMAARTVCPPHKVELEALLEKQAYKEQVLAKRLTMLELLEKYPALRPPIDHLCELLPRLQARYYSIASSSKVHPNSVHICAVVVEYETKAGRINKGVATNWLRAKEEGALVPMFVRKSQFRLPFKATTPVIMVGPGTGVAPFIGFIQERAWLRQQGKEVGETLLYYGCRRSDEDYLYREELAQFHRDGALTQLNVAFSREQSHKVYVQHLLKQDREHLWKLIEGGAHIYVCGDARNMARDVQNTFYDIVAELGAMEHAQAVDYIKKLMTKGRYSLDVWS
